# Serious postoperative complications induced by medical glue: three case reports

**DOI:** 10.1186/s12876-019-1142-6

**Published:** 2019-12-21

**Authors:** Wei Li, Mingming Xiao, Yujia Chen, Jiaxing Yang, Donghui Sun, Jian Suo, Daguang Wang

**Affiliations:** Department of Gastrointestinal Surgery, First Hospital of Jilin University, Changchun, 130021 Jilin China

**Keywords:** Medical glue, Foreign body, Medical biological glue, Complications, Case report

## Abstract

**Background:**

Various types of medical glues/adhesives/topical coagulants’ (referred to as MG hereinafter) have widespread application as surgical adhesives, and have been shown to be safe and effective for a broad range of usage, such as in hemostasis, reinforcement of intestinal anastomoses or sites of potential fluid leakage, adhesion of two surfaces, wound closure, and vascular embolization. However, inappropriate application of MG may sometimes lead to serious complications. Herein, we describe three cases of serious postoperative complications induced by a possible inappropriate use of N-butyl-2-cyanoacrylate MG (NBCA MG).

**Case presentation:**

Three patients presented with abdominal pain (chronic pain in cases 1 and 2, and acute pain in Case 3), hematochezia (Case 2), and intestinal obstruction (Case 3). All patients had a history of abdominal surgery and intraoperative use of NBCA MG. Abdominal computed tomography and gastroenterological endoscopy revealed foreign bodies (solidified MG in cases 1 and 2) and intestinal obstruction related to a mass of residual non-absorbed MG causing an internal hernia from a dense adhesion (Case 3). All patients underwent exploratory laparotomy, which revealed duodenal perforation, colonic erosion, and an internal hernia, all of which was related to MG use. We undertook removal of the foreign bodies (cases 1 and 2), surgical closure of the site of duodenal erosion (Case 1), partial colectomy (Case 2), and partial enterectomy (Case 3).

**Conclusion:**

Inappropriate application of MG may induce serious complications. We emphasize the importance of careful evaluation of the indications, dosage, and spraying thickness of MG in clinical practice. Serious complications caused by inappropriate application of MG should be reported to raise awareness in the surgical fraternity.

## Background

In surgical practice, various forms of topical medical glues (MG) have widespread application as an adhesive or a topical anticoagulant [1]. The safety and efficacy of MG across a broad spectrum of usage, such as in hemostasis, reinforcement of intestinal anastomoses or sites of potential fluid leakage, adhesion of two surfaces, wound closure, and vascular embolization, is validated by a large evidence base in the literature [2–6]. For example, absorbable hemostatic gauze used with MG was shown to prevent massive presacral hemorrhage during total mesorectal excision [2]. N-butyl-2-cyanoacrylate MG (NBCA MG) is effective and safe for the fixation of totally extraperitoneal prostheses [3]. Moreover, tissue coverage with polyglycolic acid sheets and fibrin glue appeared to be effective in minimizing the occurrence of a delayed duodenal perforation after endoscopic resection [4]. Embolization induced by the FuAiLe MG was shown to effectively control active bleeding from peripheral renal arteries in patients with hemorrhagic urological emergencies [5].

However, the use of MGs may induce serious iatrogenic complications. The use of biological glue for hemostasis in a friable right atrium subsequently led to the formation of a right atrial mass that was composed of unabsorbed and infected collection of the biological glue [6]. Another case report showed acute limb ischemia was induced by embolization that resulted from the distal embolization of biological glue 45 days after surgical repair of an 8 cm-long ascending aortic aneurysm [7].

The complications associated with the use of MG are rare, but can be very serious. This case series describes three patients who developed serious postoperative complications, including duodenal perforation, colonic perforation, and abdominal internal hernia, at our hospital. All of these complications were attributable to the presence of foreign bodies formed by residual NBCA MG.

## Case presentation

### Case 1

**Fig. 1:**
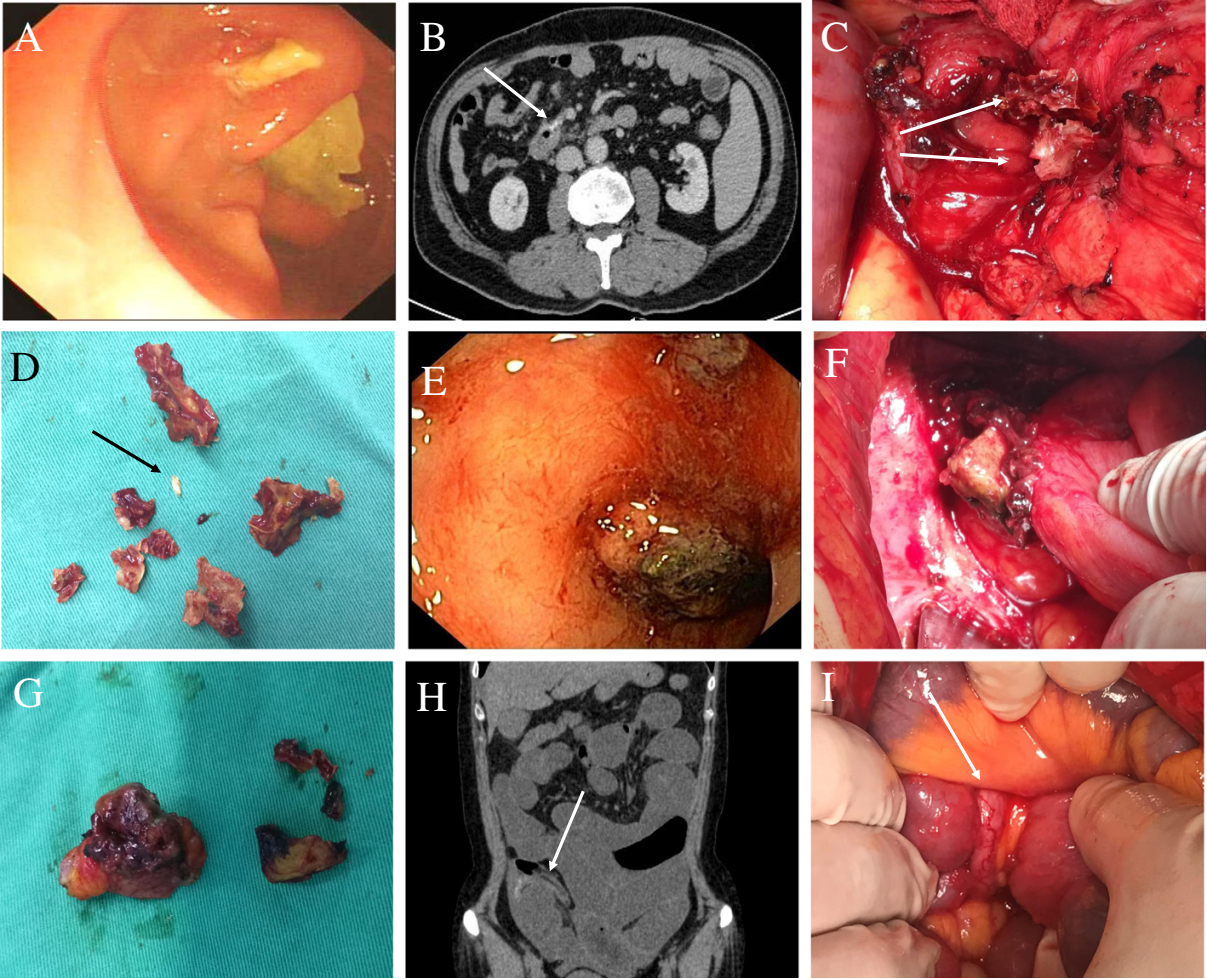
The presence of foreign bodies (medical glue). **a** Endoscopic photograph showing foreign bodies eroding the descending segment of the duodenum (Case 1). **b** Cross-sectional abdominal CT showing the duodenum (Case 1); **c** foreign bodies penetrated the duodenum (Case 1); **d** isolated foreign bodies (arrow: vascular clamp, Case 1); **e** an irregular protrusion observed on colonoscopy (Case 2); **f** foreign body penetration of the intestinal cavity (Case 2); **g** isolated foreign bodies (Case 2); **h** longitudinal-section of abdominal CT delineating the hernia (arrow: appendix, Case 3); **i** hernial ring formed by the adhesion of the appendix and the mesocolic root (arrow: appendix, Case 3)

A 68-year-old man was admitted to our hospital in May 2017 with a complaint of upper abdominal pain that had persisted for 6 months. The patient previously underwent a laparoscopic right hemicolectomy in October 2016. An upper endoscopy conducted at admission revealed flaky foreign bodies eroding into the descending segment of the duodenum, foreign bodies embedded in the anterior aspect of the duodenum, and narrowing of the duodenal canal (Fig. 1a). On abdominal computed tomography (CT), there was a striped high-density shadow on the local intestinal wall of the descending segment of the duodenum (150 HU); there was irregularity of the surface of the intestinal wall and obliteration of the demarcation between the local duodenum and adjacent small intestinal wall as well as of the fat plane between the serosa of the duodenum and the surrounding inflamed retroperitoneal tissue (Fig. 1b). The patient was diagnosed with duodenal perforation was tentatively established. Exploratory laparotomy revealed multiple hard foreign bodies, besides 3 hem-o-lock clips, between the pancreas and the descending segment of the duodenum. Those foreign bodies had penetrated the duodenum and eroded the mesentery (Fig. 1c). We removed the foreign bodies and two hem-o-lock clips, repaired the duodenum with interrupted sutures. The isolated foreign bodies were definitively identified as NBCA MG from a review of the patient’s surgical record of the previous laparoscopic radical right hemicolectomy (Fig. 1d). The postoperative period was uneventful and the patient was discharged 8 days post the surgery, when oral intake was successfully resumed.

### Case 2

In May 2017, a 49-year-old female patient presented with complaints of abdominal pain of 2 years’ duration and hematochezia for the past 6 months. The patient had previously undergone a left oophorectomy in November 2014 because of an ovarian cyst, and NBCA MG was used in the course of the operation. Colonoscopy after hospital admission revealed a 1.5 × 1.5 cm^2^ irregular mass, situated approximately 50 cm from the anus, that protruded into the lumen and was surrounded by a hard, dark brown substance (Fig. 1e). Abdominal CT revealed inhomogeneous thickening of the intestinal wall at the colosigmoid junction, narrowing of the colonic lumen, loss of distinct demarcation of the local serosal surface from that of the adjacent surface of the uterus, and surrounding fat place was lost. The patient was diagnosed with colonic erosion, and exploratory laparotomy revealed hard foreign bodies that penetrated into the intestinal cavity through the intestinal wall (Fig. 1f). We undertook a partial colectomy together with a primary colo-colostomy. The foreign bodies extracted from this patient were identified as NBCA MG (Fig. 1g). The patient recovered uneventfully; she was discharged 7 days postoperatively after she was ascertained to have no abdominal pain.

### Case 3

A 51-year-old woman was hospitalized in January 2018 with the chief complaints of abdominal pain for a duration of 5 days and failure to pass flatus and stool for 4 days. The patient had previously undergone laparoscopic radical resection for cancer of the sigmoid colon in October 2017; NBCA MG was used to “close” the hiatus between the mesentery and the posterior peritoneum. Abdominal CT at admission revealed small intestinal dilation and fluid distension in the middle and lower abdomen as well as narrowing of the ileocolic region (Fig. 1h). The patient was diagnosed with an abdominal internal hernia together with intestinal obstruction and peritonitis. An exploratory laparotomy revealed adhesion of the small intestine to the root of the mesocolon and the formation of a hernial ring, created by the adhesion of the appendix to the mesocolic root (Fig. 1i). Moreover, partial incarceration of the small intestine into the hernial ring (situated 20 cm from the ileocecus) had caused intestinal necrosis. On adhesiolysis, we observed hard foreign bodies at the root of the mesocolon. Subsequently, we conducted a small bowel resection and primary enteroenterostomy. The postoperative period was uneventful, and the patient was discharged 7 days after the surgery, after establishing that she was free of abdominal pain and had resumed normal bowel movement.

## Discussion and conclusion

There are great advantages associated with the use of MG as an adhesive and a hemostatic agent using appropriate indications [8]. However, the improper application of MG may lead to serious complications. Herein, we describe three patients who experienced postoperative complications induced by residual NBCA MG.

In Case 1, massive hemorrhage following extensive dissection during a laparoscopic radical right hemicolectomy necessitated the use of NBCA MG after hem-o-lock clips failed to achieve hemostasis; however, the NBCA MG did not postoperatively degrade in time, probably because of excessive quantities used. The foreign bodies thus formed by the residual NBCA MG directly contributed to chronic duodenal perforation.

In Case 2, NBCA MG was used for hemostasis after a left oophorectomy. Because of what appears to be excessive application of NBCA MG, the glue did not degrade postoperatively even after 30 months. The surgical site in Case 2 was situated close to the descending and sigmoid colons, and the foreign bodies formed by residual NBCA MG directly caused a colon perforation.

In Case 3, the inappropriate and possibly excessive use of MG to “close” an operative mesenteric defect by adhesion of the mesentery and posterior peritoneum during radical resection of cancer of the sigmoid colon. The use of MG appears to have caused adhesions of adjacent normal free tissues and, thus, directly contributed to the occurrence of an abdominal internal hernia.

The occurrence of MG-induced complications was closely associated with the location, dosage, and spraying thickness of MG. Therefore, indications for use of MG should be carefully evaluated, especially in the context of local tissues. Excessive application of MG appears to be able to lead to the formation of hard foreign bodies that may adhere to and erode into surrounding tissues and hollow organs like the gut. Most types of MG are amenable to gradual degradation, absorption, and excretion in vitro; however, special attention is needed to the amount of MG used during surgery. To avoid the formation of foreign bodies in vivo and to ensure optimal results, MG should be applied as little, thin, and even as possible.

To summarize, we described cases of duodenal perforation, colonic perforation, and abdominal internal hernia induced by residual NBCA MG. To avoid serious postoperative complications, the indications, dosage, and spraying thickness of MG should be carefully evaluated prior to its intraoperative use.

## Data Availability

All data sets supporting the findings and inferences reported in this article are included within the article.

## Abbreviations

CT: Computed tomography
NBCA MG: N-butyl-2-cyanoacrylate medical glue
